# PD-L1 and PD-1 expression are correlated with distinctive clinicopathological features in papillary thyroid carcinoma

**DOI:** 10.1186/s13000-017-0662-z

**Published:** 2017-10-03

**Authors:** Yanhua Bai, Dongfeng Niu, Xiaozheng Huang, Ling Jia, Qiang Kang, Fangyuan Dou, Xinqiang Ji, Weicheng Xue, Yiqiang Liu, Zhongwu Li, Qin Feng, Dongmei Lin, Kennichi Kakudo

**Affiliations:** 1 Key Laboratory of Carcinogenesis and Translational Research (Ministry of Education), Department of Pathology, Peking University Cancer Hospital & Institute, No. 52 Fucheng Road, Haidian District, Beijing, 100142 China; 2 Key Laboratory of Carcinogenesis and Translational Research (Ministry of Education), Department of Medical Statistics, Peking University Cancer Hospital & Institute, No. 52 Fucheng Road, Haidian District, Beijing, 100142 China; 3 Department of Pathology, Nara Hospital, Kindai University Faculty of Medicine, Ikoma-city, Nara, 630-0293 Japan

**Keywords:** BRAF V600E, Chronic lymphocytic thyroiditis, Papillary thyroid carcinoma, PD-1, PD-L1, Tumor-infiltrating lymphocytes

## Abstract

**Background:**

Immune checkpoint blockade targeting PD-1/PD-L1 has shown efficacy in several types of cancers. However, the correlation between PD-L1/PD-1 expression and the specific clinicopathological features in papillary thyroid carcinoma (PTC) has not been investigated.

**Methods:**

We examined the immunohistochemical expression of PD-L1, PD-1, and BRAF V600E on whole-tissue sections from 126 cases of primary PTC more than 1 cm in size. The correlation between the PD-L1/PD-1 expression and the clinicopathological features was evaluated*.*

**Results:**

PD-L1 was positively expressed in 53.2% PTCs, and its expression was positively correlated with rich tumor-infiltrating lymphocytes (TILs), background chronic lymphocytic thyroiditis (CLT), female gender, absence of psammoma bodies, and PD-1 expression. Among these parameters, rich TILs, female gender, and absence of psammoma bodies were independent factors affecting PD-L1 expression on the multivariate logistic regression analysis. PD-1 expression was detected in the TILs and was positively correlated with rich TILs, background CLT, and absence of stromal calcification. Lack of stromal calcification was an independent factor affecting PD-1 expression. Neither PD-L1 nor PD-1 expression showed significant correlation with BRAF V600E expression.

**Conclusions:**

Our results show that the distinctive pathological features of PTCs, including TILs, background CLT, female gender, psammoma bodies, and stromal calcification, are useful parameters for predicting PD-L1 or PD-1 expression.

## Background

PTC is the most common malignancy of the endocrine organs, and its incidence has increased in the past decade. Although PTC overall has a good prognosis, more than 10% of patients with PTC develop recurrence or distant metastasis after surgery, and some of these patients with succumb to PTC [1]. Although the molecular mechanisms responsible for the initiation and progression of PTC are not fully understood, the mitogen-activated protein kinase (MAPK) pathway has been implicated in the development of more than 70% of PTCs, as well as BRAF and RAS mutations and gene fusions involving the RET and NTRK1 tyrosine kinase [2–4]. The BRAF mutations play an important role in this process, and the BRAF V600E is the most common alteration. In recent years, a novel mutation-specific antibody targeting BRAF V600E protein has become commercially available. Its specificity and sensitivity to identify the BRAF V600E gene mutation have been verified in many series of tumors, including melanoma and PTC, and it could be used as a surrogate of genetic detection of BRAF V600E mutation [5–7]. This has made it feasible to detect BRAF V600E mutation in PTC using immunohistochemical staining.

As the largest endocrine organ in humans, the thyroid is frequently affected by organ-specific and systemic autoimmune diseases, including chronic lymphocytic thyroiditis (CLT), Graves’ disease, and Riedel’s thyroiditis. Among them, the most frequent condition associated with PTC is chronic lymphocytic thyroiditis [8, 9]. Many studies have indicated that up to one-third of PTCs arise in the setting of chronic lymphocytic thyroiditis, although these studies tend to lack serologic proof of preexisting thyroiditis [9]. In recent years, immune checkpoint blockade targeting PD-1/PD-L1 has shown efficacy in several types of cancers, and immunohistochemical expression of PD-L1 in cancer has shown significant correlation with clinical outcomes [10–15]. PD-L1, also known as CD274, is a cell-surface glycoprotein of the B7 family. It is expressed on various tissues including solid tumors, and it can facilitate immune evasion and T cells exhaustion. The interaction of PD-1 on the surface of T cells with PD-L1 on tumor cells suppresses TCR-mediated proliferation and activation, and inhibits T cell cytolysis. As a result, increased PD-L1 expression by cancer cells is a fundamental host immune escape mechanism [16]. These findings prompted us to examine the relationship between PD-L1/PD-1 expression and a background of CLT in PTC.

The purpose of the present study is to answer the following questions: (1) What is the correlation between the PD-L1/PD-1 expression and the clinicopathological parameters, including a background of CLT in PTCs? (2) What is the relationship between the PD-L1/PD-1 expression and the BRAF V600E expression? (3) What clinicopathological factors affect PD-L1/PD-1 expression in PTCs?

## Methods

### Patients and samples

One hundred twenty-six (126) consecutive patients with a primary PTC measuring more than 10 mm in diameter who underwent surgical treatment in 2013 at Peking University Cancer Hospital, Beijing, China, were enrolled, including 96 females and 30 males. The patients’ age at surgery was 43.94 ± 13.15 (mean ± s.d.). All patients had no evidence of distant metastasis at the time of surgery. All histological subtypes of PTCs were included except microcarcinoma [17].

Sections stained with hematoxylin and eosin from the 126 cases of PTC were histologically evaluated by two pathologists, BY and ND, to confirm the histological subtypes and to examine TILs, psammoma bodies, stromal calcification, bone formation within the tumor mass, and extrathyroid invasion, multifocality as well as background disease. Of these 126 PTCs, 114 cases were conventional type, 8 were follicular variants, 2 were solid variants, 1 was tall cell variant, and 1 case was a Warthin-like tumor variant.

### Definition of the Clinicopathological features

The evaluation of TILs in PTC was according to the evaluation of TILs in breast cancer recommended by an International TILs Working Group in 2014 [18]. Most PTCs have a small number of TILs. In the present study, if TILs occupied 10% or more of the tumor stroma, it was considered TILs-rich, otherwise it was considered TILs-poor. The definitions of psammoma bodies, stromal calcification, and bone formation were described in detail in our earlier study [19].

**Fig. 1:**
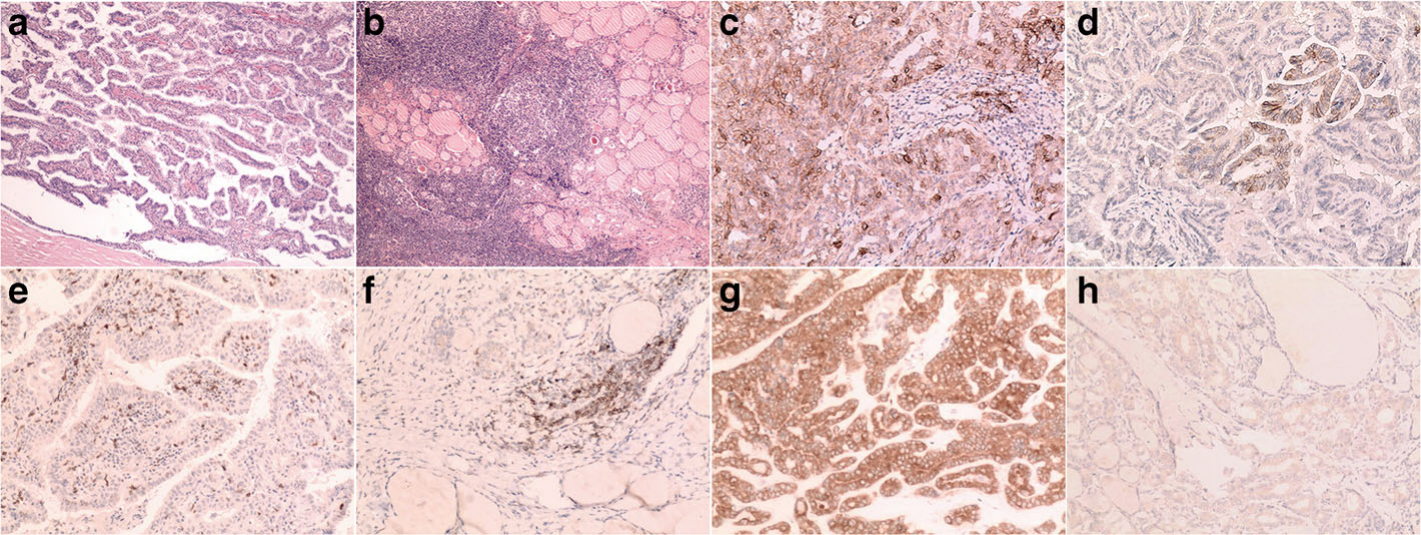
**a**, Papillary thyroid carcinoma (PTC) with predominant papillary structures; **b**, The background thyroid tissue of (**a**), which showed dense lymphoplasmacytic infiltrates and germinal center formation indicating the presence of chronic lymphocytic thyroiditis; **c**, PD-L1 was diffusely expressed both on the cell membrane and the cytoplasm. Please note there was also staining on the tumor-infiltrating lymphocytes (TILs), which should not be regarded as positive staining of the tumor cells; **d**, PD-L1 was focally expressed on the tumor cell membrane; **e**, PD-1 expression of the TILs was identified in the tumor stroma; **f**, PD-1 expression was noted in the tumor invasive front; **g**, BRAF V600E was strongly and diffusely expressed on the tumor cytoplasm; **h**, Mild but diffuse expression of BRAF V600E was noted on the PTC

The background disease was classified as CLT and non-CLT in the present study. According to Mizukami’s definition, CLT was divided into four groups: chronic thyroiditis, oxyphilic; chronic thyroiditis, mixed; chronic thyroiditis, hyperplastic; and chronic thyroiditis, focal [20]. The first group demonstrated classic Hashimoto’s disease histology [21]. The second group showed a less lymphoplasmacytic infiltrate than the former, with minimal fibrosis. The third and fourth groups have only a small or focal lymphocytic reaction. Tumor-bearing thyroid tissues demonstrating characteristics of the first and second groups were categorized as CLT in the present study (Fig. 1a and b), and those that did not meet the diagnostic criteria of CLT were categorized as non-CLT, including the third and fourth group in Mizukami’s definition, since the latter more likely represented types of non-specific thyroiditis other than autoimmune thyroiditis.

The pT, pN, and stage groupings were categorized according to the cancer staging of the seventh edition of American Joint Committee on Cancer.

### Immunohistochemical analysis

Formalin-fixed, paraffin-embedded (FFPE) whole-tissue sections were cut to generate 4-μm unstained slides in 126 PTCs, including the tumor center and tumor invasive front, for PD-L1, PD-1, and BRAF V600E staining. All stained sections were examined microscopically and scored independently by three pathologists (BY, ND, and XW).

PD-L1 staining was performed with an anti-human PD-L1 rabbit monoclonal antibody at working solution (SP142; Zhongshan Golden Bridge Biotechnology, Beijing, China) on an automated staining platform (BOND-III, Leica Biosystems Ltd., Newcastle, UK) using a Bond Polymer Refine Detection kit (Leica Biosystems Ltd., Newcastle, UK) according to the manufacturer’s instructions. To verify the specificity of the immunostaining, primary antibody was replaced by phosphate-buffered saline from the staining procedure in a set of control sections. Patterns with 5% or greater positive tumor cells were considered positive, and those with less than 5% positive cells were considered negative [15].

For PD-1, the FFPE sections were deparaffinized and rehydrated, and then antigen retrieval was carried out in a PTLink machine (Dako, Glostrup, Denmark). Endogenous peroxidase activity was blocked by incubation in 3% H_2_O_2_ solution at room temperature for 10 min. Then the sections were incubated with mouse anti-PD-1 monoclonal antibody at working solution (UMAB199; Zhongshan Golden Bridge Biotechnology, Beijing, China) at 37 °C for 15 min, followed by incubation with anti-mouse IgG-HRP conjugate (EnVision FLEX/HRP; Dako, Glostrup, Denmark) at room temperature for 25 min. Antibody binding was visualized using an EnVision FLEX DAB CHROMOGEN kit (Dako, Glostrup, Denmark) according to the manufacturer’s instructions. For general negative controls, the primary antibodies were replaced by phosphate-buffered saline. Positive immunostaining of PD-1 was regarded as staining on ≥3 TILs per high power field (Olympus, BX43) in the hot spot inside the tumor or in the tumor invasive front.

BRAF V600E staining was performed using the anti-BRAF V600E mouse monoclonal primary antibody at working solution (VE1; Ventana Medical Systems, Oro Valley, AZ, USA) on the Benchmark XT platform (Ventana Medical Systems). The specific visualization of V600E-mutated BRAF protein was accomplished using the ultraView Universal DAB Detection Kit (Ventana Medical Systems). Pre-validated BRAF V600E positive and negative PTC specimens were used as positive and negative controls, respectively. According to previous studies together with our experience, the immunoreactivity of V600E-mutated BRAF protein was evaluated as positive if unequivocal diffuse cytoplasmic staining of tumor cells was identified [5, 6, 22]. The staining intensities included weak, moderate, and strong. Focal staining, any type of isolated nuclear staining, and weak staining of single interspersed cells were scored as negative.

### Statistical analysis

Time-independent and categorical data were evaluated using the chi-squared test, continuity correction, or Fisher’s exact probability test as appropriate. The multivariate logistic regression model was used to evaluate the multivariate analyses affecting PD-L1 and PD-1 expression. The differences were considered significant when the probability (*P*-value) was less than 0.05. Data analysis was performed using SPSS 17.0 statistical software.

## Results

### Clinicopathological significance of PD-L1, PD-1, and background of CLT

PD-L1 expression was located in the membrane with or without cytoplasmic staining. The staining pattern was diffuse or focal (Fig. 1c and d). Two of the eight follicular variant PTCs showed focal positive staining of PD-L1. One of the two solid variant PTCs and the only case of Warthin-like tumor demonstrated diffuse staining of PD-L1. That case of tall cell variant showed negative PD-L1 staining. Positive PD-L1 expression was identified in 53.2% PTCs, and positive PD-L1 expression was significantly correlated with rich TILs, female gender, absence of psammoma bodies, and a background of CLT (Table 1).

**Table 1:** The correlation between the PD-L1 expression and other clinicopathological parameters

	PD-L1 expression		*P*-value
	+ (53.2%)	- (46.8%)	
Age			
< 45 years	33	33	0.454
≥ 45 years	34	26	
Gender			
Female	58	38	0.004
Male	9	21	
Tumor size			
≤ 2 cm	44	39	0.147
> 2 and ≤4 cm	19	20	
> 4 cm	4	0	
Extrathyroid invasion			
-	15	15	0.690
+	52	44	
pT			
1b	12	13	0.699
2	4	2	
3	51	44	
pN			
0	25	22	0.186
1a	30	19	
1b	12	18	
Stage grouping			
I + II	8	8	0.294
III	47	34	
IVA	12	17	
Psamomma body			
-	39	19	0.003
+	28	40	
Stromal calcification			
-	38	27	0.220
+	29	32	
Bone formation			
-	66	57	0.911
+	1	2	
Multifocality			
Unifocal	50	38	0.212
Multifocal	17	21	
TILs			
Poor	39	54	0.001
Rich	28	5	
Background disease			
CLT	21	6	0.004
Non-CLT	46	53	

*pT* pathological tumor category, *pN* pathological lymph node category, *TILs* tumor-infiltrating lymphocytes, *CLT* chronic lymphocytic thyroiditis

PD-1 expression was identified in the lymphocytes both within the cancer and in the non-neoplastic thyroid tissue including CLT. Only PD-1 expression on the TILs both inside of and in the invasive front of PTC was counted (Fig. 1e and f). PD-1 was expressed in 84.9% PTC cases. Expression of PD-1 was significantly correlated with rich TILs, absence of stromal calcification and presence of background CLT (Table 2).

**Table 2:** The correlation between the PD-1 expression and other clinicopathological parameters

	PD-1 expression		*P*-value
	+ (84.9%)	- (15.1%)	
Age			
< 45 years	56	10	0.981
≥ 45 years	51	9	
Gender			
Female	81	15	0.989
Male	26	4	
Tumor size			
≤ 2 cm	71	12	0.136
> 2 and ≤4 cm	34	5	
> 4 cm	2	2	
Extrathyroid invasion			
-	27	3	0.550
+	80	16	
pT			
1b	22	3	0.477
2	6	0	
3	79	16	
pN			
0	41	6	0.350
1a	43	6	
1b	23	7	
Stage grouping			
I + II	16	0	0.095
III	69	12	
IVA	22	7	
Psamomma body			
-	49	9	0.899
+	58	10	
Stromal calcification			
-	61	4	0.004
+	46	15	
Bone formation			
-	105	18	0.938
+	2	1	
Multifocality			
Unifocal	77	11	0.218
Multifocal	30	8	
TILs			
Poor	74	19	0.011
Rich	33	0	
Background disease			
CLT	27	0	0.030
Non-CLT	80	19	

*pT* pathological tumor category, *pN* pathological lymph node category, *TILs* tumor-infiltrating lymphocytes, *CLT* chronic lymphocytic thyroiditis

The presence of CLT usually indicates autoimmune disease of the thyroid, although it may overlap with non-specific lymphocytic thyroiditis. The presence of CLT was significantly correlated with both PD-L1 and PD-1 expression. PD-L1 expression was in 77.8 and 46.5% cases of PTCs with and without CLT in the background thyroid tissue, respectively (*P* = 0.004, Table 1). PD-1 expression was in 100 and 80.8% PTCs with and without CLT in the background thyroid tissue, respectively (*P* = 0.030, Table 2). The presence of CLT was also correlated with negative extrathyroid invasion, low stage grouping, and rich TILs (*P* = 0.020, 0.032, and 0.001, respectively, Table 3).

**Table 3:** The correlation between the background disease and other clinicopathological parameters

	Background disease		*P*-value
	CLT	Non-CLT	
Age			
< 45 years	14	52	0.950
≥ 45 years	13	47	
Gender			
Female	24	72	0.081
Male	3	27	
Tumor size			
≤ 2 cm	18	65	0.561
> 2 and ≤4 cm	9	30	
> 4 cm	0	4	
Extrathyroid invasion			
-	11	19	0.020
+	16	80	
pT			
1b	9	16	0.089
2	2	4	
3	16	79	
pN			
0	11	36	0.203
1a	13	36	
1b	3	27	
Stage grouping			
I + II	7	9	0.032
III	17	64	
IVA	3	26	
Psamomma body			
-	10	48	0.290
+	17	51	
Stromal calcification			
-	16	49	0.368
+	11	50	
Bone formation			
-	27	96	0.839
+	0	3	
Multifocality			
Unifocal	19	69	0.946
Multifocal	8	30	
TILs			
Poor	5	88	0.001
Rich	22	11	

*pT* pathological tumor category, *pN* pathological lymph node category, *TILs* tumor-infiltrating lymphocytes, *CLT* chronic lymphocytic thyroiditis

### Correlation between PD-L1, PD-1, and BRAF V600E expression

**Fig. 2:**
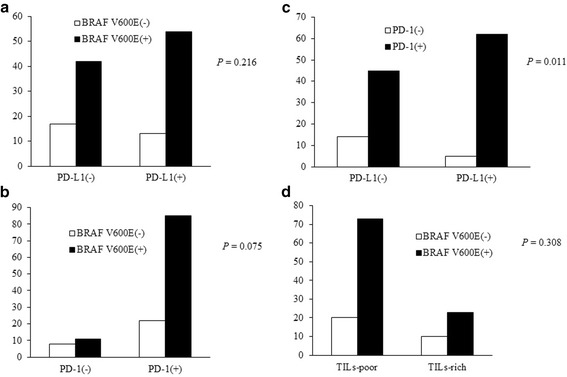
**a**, The correlation between BRAF V600E and PD-L1 expression; **b**, The correlation between BRAF V600E and PD-1 expression; **c**, The correlation between PD-L1 and PD-1 expression; **d**, the correlation between BRAF V600E expression and tumor-infiltrating lymphocytes (TILs)

Positive BRAF V600E staining (Fig. 1g and h) was seen in 76.2% of 126 cases of PTCs. BRAF V600E staining was not correlated with PD-L1 or PD-1 expression (Fig. 2a and b). PD-L1 and PD-1 expression were significantly correlated with each other (*P* = 0.011, Fig. 2c).There was no significant correlation between BRAF V600E expression and TILs (Fig. 2d).

### Clinicopathological features affecting PD-L1 and PD-1 expression

Clinicopathological features that are correlated with PD-L1 expression including rich TILs, a background disease of CLT, female gender, and the absence of psammoma bodies, together with PD-1 expression, were subsequently analyzed using the multivariate logistic regression analysis model. Among them, rich TILs, female gender, and the absence of psammoma bodies, were independent factors to predict PD-L1 expression (Table 4). Rich TILs, a background disease of CLT, absence of stromal calcification, together with PD-L1 expression were analyzed using the multivariate logistic regression analysis model, to detect independent factors predicting PD-1 expression. Among them, only the absence of stromal calcification was independent predicting factor for PD-1 expression (Table 5).

**Table 4:** Multivariate logistic analysis of examined clinicopathological parameters influencing PD-L1 expression

Parameter	OR	95% CI	*P*- value
Gender			
Male	1.000	/	0.019
Female	3.393	1.225–9.395	
Background disease			
Non-CLT	1.000	/	0.803
CLT	1.206	0.276–5.265	
Psammoma body			
-	1.000	/	0.002
+	0.263	0.112–0.614	
PD-1 expression			
-	1.000	/	0.067
+	3.099	0.923–10.400	
TILs			
Poor	1.000	/	0.028
Rich	5.036	1.189–21.340	

*OR* odds ratio, *CI* confidence interval, *CLT* chronic lymphocytic thyroiditis, *TILs* tumor-infiltrating lymphocytes

**Table 5:** Multivariate logistic analysis of examined clinicopathological parameters influencing PD-1 expression

Parameter	OR	95% CI	*P*- value
Background disease			
Non-CLT	1.000	/	0.998
CLT	9.155E7	/	
Stromal calcification			
-	1.000	/	0.020
+	0.237	0.070–0.799	
PD-L1 expression			
-	1.000	/	0.115
+	2.542	0.796–8.120	
TILs			
Poor	1.000	/	0.998
Rich	4.455E7	/	

*OR* odds ratio, *CI* confidence interval, *CLT* chronic lymphocytic thyroiditis, *TILs* tumor-infiltrating lymphocytes

## Discussion

In this study, we examined the expression of PD-L1 and PD-1 in 126 cases of PTCs and analyzed their association with clinicopathological parameters. Both PD-L1 and PD-1 expression were correlated with CLT in the background thyroid tissue. Many studies have indicated that up to one-third of PTCs arose in a background of chronic thyroiditis. However, these studies did not have serologic evidence of preexisting thyroiditis [9]. In our cases, 21.4% of PTCs occurred in the thyroid with a background of CLT, which was less frequent than previously reported. The discrepancy between ours and those previously reported was most likely due to the fact that small or focal lymphocytes infiltration was not regarded as CLT in our study, since they most likely represent non-specific thyroiditis other than autoimmune thyroiditis. Because PTC and thyroiditis are both common conditions, the coexistence of them is more likely to be a coincidental incident than an etiologic relationship [8, 9]. For patients with CLT, T lymphocytes were of predominantly suppressor type within the thyroid, whereas there were mostly helper T cells in the periphery blood [23, 24]. A previous study has indicated that positive thyroglobulin antibody in the serum of PTC patients was associated with lower tumor stage on univariate analysis and associated with a favorable clinical outcome [25, 26], which was similar to our present finding (PTC with a background of CLT correlated with lower tumor stage and negative extra-thyroidal invasion). Both PD-L1 and PD-1 were highly expressed in PTC cases harboring a background CLT in our study, although the presence of a CLT background was not an independent factor affecting PD-L1 or PD-1 expression. It should be noticed that the presence of CLT may possibly influence the efficacy of immunotherapy using antibodies against PD-L1/PD-1, because of its intimate correlation with both PD-1 and PD-L1 expression. Further studies are required to answer this question.

Expression of PD-L1 and that of PD-1 were significantly correlated each other in the present study, and PD-1 expression may influence the clinical significance of PD-L1 expression, and vice versa. A previous study has classified the tumor microenvironments into four groups based on PD-L1 expression and TILs [27]. Different immunotherapy strategies should be applied in different groups [27]. The expression status of PD-1 on the TILs is a more direct indicator than TILs itself to reflect the tumor immune suppression status, and thus clarifying the PD-L1 and PD-1 expression status of a tumor could help to select proper tumor immunotherapy approaches.

Recently, high expression of PD-L1 was reported by Chowdhury et al. to be correlated with invasiveness of PTC and poor disease-free survival [28]. Our cases were all PTCs with tumor size >1 cm, and of our 126 cases, 124 showed an invasive growth pattern. Extrathyroid invasion is a useful parameter to predict tumor recurrence or tumor death [1]. However, the correlation between expression of PD-L1 and extrathyroid invasion did not reach a statistical significance both in Chowdhury’s research and in our study [28]. Although we did not examine the direct correlation between PD-L1 expression and disease-free survival, the clinical correlation of PD-L1 expression did not suggest a possible poor clinical outcome in PD-L1 positive cases. Expression of PD-L1 was correlated with the female gender and the absence of psammoma bodies, and expression of PD-1 was correlated with the absence of stromal calcification. Psammoma bodies were considered as being formed by calcification subsequent to the necrosis of tumor thrombi [29]. Carcangiu et al. reported that PTC patients with psammoma bodies were affected more often by persistent disease and had a higher incidence of lymph node and pulmonary metastasis [30]. Our previous study indicated that PTC patients with psammoma bodies, compared to those without, had poorer disease-free survival [19]. The significant correlation between PD-L1 expression and absence of psammoma bodies may indicate that PD-L1 expression is possibly associated with a favorable clinical outcome. The stromal calcification was regarded as being formed due to the deposition of calcium phosphate in the fibrous stroma. The formation of both psammoma bodies and stromal calcification requires some time and may indicate a relatively long clinical course. The positive correlation between PD-L1 expression and the absence of psammoma bodies, and between PD-1 expression and the absence of stromal calcification, may indicate that both PD-L1 and PD-1 expressions are correlated with a short survival clinical course, and they are probably involved in the tumor growth, as indicated in ovarian cancer [31]. Ahn recently used tissue microarrays to investigate 407 primary thyroid cancers for PD-L1 expression, and no significant association was found between PD-L1 expression and clinicopathologic variables, disease progression, and oncogenic mutation [32], which was different from our finding. Further studies using whole-tissue sections are required to confirm or disprove the clinical correlations of PD-L1 and PD-1.

The constitutively active BRAF V600E protein has been associated with worse clinical outcomes in thyroid cancer [33]. Angell et al. reported that BRAF V600E was associated with increased PD-L1 expression in PTC [34]. In our study, the correlation between PD-L1 and BRAF V600E did not reach a statistical significance. This could be explained partly by the sample selection. In Angell et al.’s 33 cases of PTC, 78.8% were pT1 or pT2 cases and their cases in Stage I totaled 60.7%. However, our 126 cases of PTC were consecutive cases, with 19.8% of pT1 and 12.7% of Stage I and II. Another parameter affecting the correlation between PD-L1 and BRAF V600E was the presence of a background CLT. After excluding PTC cases with a background of CLT, BRAF V600E was positively correlated with PD-L1 expression (manuscript in progress).

## Conclusion

We examined the expression of PD-L1, PD-1, and BRAF V600E in 126 cases of PTC, and found that distinctive pathological features in PTC, including TILs, a background of CLT, psammoma bodies, and stromal calcification, were useful parameters for predicting PD-L1 or PD-1 expression, which was a novel finding and should be highlighted in the forthcoming era of immunotherapy. Further studies are necessary to determine the prognostic and therapeutic value of these findings in PTCs.

## Abbreviations

CI: Confidence interval
CLT: Chronic lymphocytic thyroiditis
FFPE: Formalin-fixed, paraffin-embedded
OR: Odds ratio
pN: Pathological lymph node category
pT: Pathological tumor category
PTC: Papillary thyroid carcinoma
TILs: Tumor-infiltrating lymphocytes
